# JKAMP inhibits the osteogenic capacity of adipose-derived stem cells in diabetic osteoporosis by modulating the Wnt signaling pathway through intragenic DNA methylation

**DOI:** 10.1186/s13287-021-02163-6

**Published:** 2021-02-12

**Authors:** Shuanglin Peng, Sirong Shi, Gang Tao, Yanjing Li, Dexuan Xiao, Lang Wang, Qing He, Xiaoxiao Cai, Jingang Xiao

**Affiliations:** 1 Department of Oral and Maxillofacial Surgery, The Affiliated Stomatology Hospital of Southwest Medical University, Luzhou, 646000 China; 2 State Key Laboratory of Oral Diseases, West China Hospital of Stomatology, Sichuan University, Chengdu, 610041 China; 3 National Key Clinical Specialty, The Affiliated Hospital of Southwest Medical University, Luzhou, 646000 China; 4 Orofacial Reconstruction and Regeneration Laboratory, The Affiliated Stomatology Hospital of Southwest Medical University, Luzhou, 646000 China; 5 Department of Oral Implantology, The Affiliated Stomatology Hospital of Southwest Medical University, Luzhou, 646000 China

**Keywords:** DNA methylation, JKAMP, Wnt signaling pathway, Adipose-derived stem cells, Osteogenic differentiation, Diabetic osteoporosis

## Abstract

**Background:**

Diabetic osteoporosis (DOP) is a systemic metabolic bone disease caused by diabetes mellitus (DM). Adipose-derived stem cells (ASCs) play an important role in bone regeneration. Our previous study confirmed that ASCs from DOP mice (DOP-ASCs) have a lower osteogenesis potential compared with control ASCs (CON-ASCs). However, the cause of this poor osteogenesis has not been elucidated. Therefore, this study investigated the underlying mechanism of the decline in the osteogenic potential of DOP-ASCs from the perspective of epigenetics and explored methods to enhance their osteogenic capacity.

**Methods:**

The expression level of JNK1-associated membrane protein (JKAMP) and degree of DNA methylation in CON-ASCs and DOP-ASCs were measured by mRNA expression profiling and MeDIP sequencing, respectively. JKAMP small interfering RNA (siRNA) and a *Jkamp* overexpression plasmid were used to assess the role of JKAMP in osteogenic differentiation of CON-ASCs and DOP-ASCs. Immunofluorescence, qPCR, and western blotting were used to measure changes in expression of Wnt signaling pathway-related genes and osteogenesis-related molecules after osteogenesis induction. Alizarin red and ALP staining was used to confirm the osteogenic potential of stem cells. Bisulfite-specific PCR (BSP) was used to detect JKAMP methylation degree.

**Results:**

Expression of JKAMP and osteogenesis-related molecules (RUNX2 and OPN) in DOP-ASCs was decreased significantly in comparison with CON-ASCs. JKAMP silencing inhibited the Wnt signaling pathway and reduced the osteogenic ability of CON-ASCs. Overexpression of JKAMP in DOP-ASCs rescued the impaired osteogenic capacity caused by DOP. Moreover, JKAMP in DOP-ASCs contained intragenic DNA hypermethylated regions related to the downregulation of JKAMP expression.

**Conclusions:**

Intragenic DNA methylation inhibits the osteogenic ability of DOP-ASCs by suppressing expression of JKAMP and the Wnt signaling pathway. This study shows an epigenetic explanation for the reduced osteogenic ability of DOP-ASCs and provides a potential therapeutic target to prevent and treat osteoporosis.

## Background

Diabetic osteoporosis (DOP) is a serious metabolic complication of diabetes mellitus (DM) in the bone and joint system. Characterized by hyperglycemic microenvironment and systemic impairment of bone microstructure, strength, and mass, patients with DOP are prone to fractures and have difficulty in controlling diabetes, which makes treatment and rehabilitation difficult [1–3]. The incidence of DOP is about 50% among DM patients [4, 5]. DOP patients with fractures or bone defects have reduced bone healing and regeneration, which leads to poor bone healing, nonunion, and bone defects [6, 7]. Studies are investigating targets to prevent or treat DOP. Bone tissue engineering is a promising method to repair bone defects, which consists of seed cells, scaffold materials, and growth factors [8, 9]. Adipose-derived stem cells (ASCs) as seed cells have attracted widespread attention for clinical applications, including reconstruction of bone defects [10]. In our previous study, the osteogenic ability of DOP-ASCs was decreased in comparison to control ASCs (CON-ASCs). However, the regulatory mechanism of osteogenic differentiation of DOP-ASCs has not been elucidated, which has hindered their application to treatment of DOP bone fractures and defects.

DNA methylation is one of the earliest discovered DNA modification pathways. Studies have shown that DNA methylation changes the chromatin structure, DNA conformation, DNA stability, and the manner through which proteins act on DNA [11, 12]. It is generally believed that hypermethylation of gene promoters contributes to gene silencing, and DNA demethylation and hypermethylation have opposite effects [13–15]. DNA methylation is very important for the self-renewal, multi-directional differentiation ability, and aging of embryonic stem cells [16]. The occurrence of osteoporosis, osteoarthritis, and other skeletal diseases has been confirmed to be related to changes in the degree of DNA methylation in stem cells [17, 18]. Thus, investigating DNA methylation of DOP-ASCs may provide a reference for the mechanism of DOP-related bone disease. Our previous study showed that the overall DNA methylation level was significantly increased in DOP-ASCs in comparison with CON-ASCs and the Wnt/β-Catenin signaling pathway related to bone differentiation was inhibited [19]. However, the regulatory mechanism of DNA methylation in differentiation of DOP-ASCs into osteoblasts has not been reported.

JNK1-associated membrane protein (JKAMP) has been shown to be associated with JNK1 through its C-terminal domain, which increases and prolongs JNK1 activity after activation [20]. In particular, c-Jun N-terminal kinases (JNKs)-members of the mitogen-activated protein kinase superfamily play an important role in regulating various cellular processes including differentiation, apoptosis, and proliferation [21]. JNK1 as a critical protein in the Wnt-PCP signaling pathway significantly regulates its downstream signaling molecules and the canonical Wnt signaling pathway [22]. The Wnt signaling pathway is a highly conserved signaling pathway. Abnormal changes in Wnt signaling pathway markers are linked to changes in bone metabolism and the osteogenic ability of mesenchymal stem cells [23–26]. Recent study has shown that JNK1 is essential for osteoblast function in vivo and in vitro [27, 28]. Therefore, JKAMP may be associated with the regulation of the osteogenic ability of mesenchymal stem cells via JNK1 and Wnt signaling pathway. However, the possible role of JKAMP and JNK1 in decline of the osteogenic capacity of DOP-ASCs is unclear.

In this study, we isolated CON-ASCs and DOP-ASCs from C57BL/6 mice and diabetic osteoporosis C57BL/6 mice, respectively. The cells were treated with Si-*Jkamp* and a plasmid (*Jkamp*) for silencing and overexpression of JKAMP, respectively. Bisulfite-specific PCR (BSP) was used to detect the methylation degree of JKAMP. Gene function analysis was employed to assess changes in the JKAMP and the related signaling pathways. Thus, we explored the effects of JKAMP on the osteogenic ability of DOP-ASCs through the Wnt signaling pathway and evaluated the role of DNA methylation in this process.

## Methods

### Establishment of the diabetic osteoporosis animal model

C57BL/6 mice were provided by the Experimental Animal Center of the Department of Basic Medicine, Southwest Medical University. All animal procedures were reviewed and approved by the Ethics Committee of the Southwest Medical University. The animal care and anesthesia were conducted in accordance with the guidelines of the Care and Use of Laboratory Animals (Ministry of Science and Technology of China, 2006). Briefly, 4-week-old male mice were randomly divided into model (DOP) and control (CON) groups by the random number table method. The CON group was fed normal feed and the DOP group was fed high-fat and sugar feed for 4 weeks. We established the DOP animal model by injection of streptozotocin (STZ; Sigma, St Louis, USA). The two groups of mice were fasted for 12 h and then the DOP group received a single intraperitoneal injection of STZ (140 mg/kg). The CON group received a single intraperitoneal injection of citric acid-sodium citrate buffer (140 mL/kg). After the procedure, each mouse was returned to its original cage for normal feeding (room temperature, 20–25 °C; relative humidity, 60%–80%, free access to water and normal feed).

### Isolation and culture of CON-ASCs and DOP-ASCs

CON-ASCs and DOP-ASCs were obtained from subcutaneous adipose tissue of the groin in CON and DOP mice. The adipose tissue was cut into pieces and incubated in 0.075% type I collagenase (Sigma-Aldrich, St Louis, USA) for 30 min at 37 °C. The digestion was terminated with 10% α-modified Eagle’s medium (α-MEM, Hyclone, Pittsburgh, USA) containing 10% fetal bovine serum (FBS, Schaumburg, USA). The cells were centrifuged at 200 g for 5 min and the supernatant was discarded. Then, the cells were resuspended in α-MEM containing 10% FBS and 1% penicillin-streptomycin, seeded in 25-cm^2^ culture flasks, and incubated at 37 °C with 5% CO_2_. Non-adherent cells were removed by changing the medium every 2 days. At 80% confluence, the cells were passaged. Passage 3 cells were used in experiments.

### Cell transfection

JKAMP siRNA was designed and synthesized by GenePharma (Shanghai, China) for gene silencing. Cells were transiently transfected using a riboFECT CP Transfection Kit (RiboBio, Guangzhou, China) in accordance with the manufacturer’s instructions. SiRNA sequences are shown in Table 1.

**Table 1:** SiRNA sequences designed for specific gene silencing

		Sequence (5′ → 3′)
SiRNA	Sense	GCACCAUGGCAGCUAUCAUTT
	Antisense	AUGAUAGCUGCCAUGGUGCTT
SiRNA-NC	Sense	UUCUCCGAACUGGUCACGUTT
	Antisense	ACGUGACACGUUCGGAGAATT

For JKAMP overexpression, JKAMP was amplified and subcloned into the pcDNA3.1 vector. The empty pGFP3.1 vector carrying eGFP was used as a negative control. Plasmids were transfected into cells using an Auto Electroporator (Bimake, TX, USA) in accordance with the manufacturer’s instructions.

### Alizarin red and ALP staining

Alizarin red and ALP staining was used to analyze mineralized nodule formation and alkaline phosphatase activity of differentiated CON-ASCs and DOP-ASCs. CON-ASCs and DOP-ASCs were seeded on 12-well plates at 5 × 10^4^ cells per well. The medium was changed to osteogenic induction medium (Cyagen, Guangzhou, China), after CON-ASCs and DOP-ASCs were transfected with Si-*Jkamp* and plasmid (Jkamp), respectively. The medium was changed every 3 days. After 3 and 5 days of osteoinduction, the osteogenesis induction medium was discarded. Cells were washed twice with PBS and then fixed with 4% neutral buffered formalin for 30 min. ALP activity was detected using an Alkaline Phosphatase Assay Kit (Beyotime, Shanghai, China) in accordance to the manufacturer’s protocol. At 14 days after osteogenesis induction, we performed alizarin red staining (Cyagen) in accordance with the manufacturer’s protocol to assess the formation of calcium nodules. Staining was observed using the DFC 7000T system (Leica, Wetzlar, Germany). Each image was selected to save by a visual camera.

### Western blot analysis

Western blotting was used to detect the levels of JKAMP, JNK1, GSK-3β, p-GSK-3β, β-catenin, RUNX2, and OPN. Total protein was isolated from cells using a total protein extraction kit (Keygen Biotech, Nanjing, China). The protein samples were mixed with loading buffer, boiled for 5 min, separated by SDS-PAGE, and transferred to polyvinylidene fluoride membranes [29, 30]. The membranes were blocked with 5% dry skim milk in Tris-buffered saline with 0.05% (v/v) Tween-20 (TBST) for 1 h and then incubated with primary antibodies against JKAMP (NBP2-36446SS) (Novus, Littleton, USA), GAPDH (ab181602), JNK1 (ab110724), β-Catenin (ab32572), RUNX2 (ab92336) and OPN (ab8448) (Abcam, Cambridge, UK), GSK-3β (12456), or p-GSK-3β (5558) (Cell Signaling Technology, Danvers, USA) at 4 °C overnight. The membrane was washed three times with TBST and then incubated with a secondary labeled anti-rabbit or anti-mouse antibody (1:3000) for 1 h. The membrane was then washed three times with TBST and developed using an enhanced chemiluminescence detection system (Bio-Rad, Hercules, USA).

### Quantitative polymerase chain reaction

Gene expression levels after 3 and 6 days of osteogenesis induction were measured by qPCR, including JNK1-associated membrane protein (*Jkamp*), c-Jun-N-terminal-kinase-1 (*Jnk1*), cadherin-associated protein, delta 1 (*β-catenin*), runt-related transcription factor 2 (*Runx2*), and osteopontin (*Opn*). The primer sequences are shown in Table 2. Briefly, an RNeasy Plus Mini kit (Qiagen, Hilden, Germany) and genomic DNA eliminator was used to isolate and purify total RNA from cells and then cDNA was synthesized using a PrimeScript RT kit with gDNA Eraser (Takara Bio, Tokyo, Japan). qPCR was performed using a PrimeScript RT-PCR Kit (Takara Bio) with the following amplification program: denaturation at 95 °C for 30 s and then 45 cycles of 95 °C for 5 s and 60 °C for 34 s for amplification. Gene expression from was averaged and normalized against *Gapdh* [31, 32].

**Table 2:** Primer sequences for qPCR amplification of specific genes

Genes		Sequence (5′ → 3′)
*Gapdh*	Forward	GGTGAAGGTCGGTGTGAACG
	Reverse	CTCGCTCCTGGAAGATGGTG
*Jkamp*	Forward	CCAATGGCTGTCGATATTCAACC
	Reverse	CTTGGGCATACCCCACATTCT
*Jnk1*	Forward	GTGGAATCAAGCACCTTCACT
	Reverse	TCCTCGCCAGTCCAAAATCAA
*β-Catenin*	Forward	GCTGCGTGGACAATGGCTACTC
	Reverse	AGCGTCAAACTGCGTGGATGG
*Runx2*	Forward	GACTGTGGTTACCGTCATGGC
	Reverse	ACTTGGTTTTTCATAACAGCGGA
*Opn*	Forward	TCCCTCCCGGTGAAAGTGACTG
	Reverse	TCCTCGCTCTCTGCATGGTCTC

### Immunofluorescence staining

ASCs and DOP-ASCs were transfected with Si-*Jkamp* or *a Jkamp* plasmid. The cells were then incubated in osteogenic induction medium for 3 days. Subsequently, the cells were fixed with 4% paraformaldehyde at 4 °C for 30 min. After treatment with 0.5% Triton X-100 for 10 min to permeabilize the cell membrane, the cells were incubated with 5% sheep serum for 1 h and then with diluted primary antibodies against RUNX2 or OPN at 4 °C overnight. The samples were rewarmed for 30 min and then incubated with a fluorescent dye-conjugated anti-rabbit secondary antibody (1:500, Invitrogen, CA, USA) for 1 h at 37 °C. The nucleus and cytoskeleton of the cells were stained with DAPI and phalloidin, respectively. Cells were washed with PBS between each step. Finally, images were captured under a confocal laser microscope (Nikon, Tokyo, Japan).

### Statistical analysis

SPSS 19.0 software (IBM, NY, USA) was used for statistical analysis. The *t* test or one-way analysis of variance (ANOVA) was applied to the experimental data to evaluate their reliability. Each experiment was repeated at least three times. The results are expressed as the mean ± standard deviation (SD). Data were considered statistically different at *P* < 0.05.

## Results

### JKAMP, the Wnt signaling pathway, and osteogenesis-related molecules are downregulated in DOP-ASCs

**Fig. 1:**
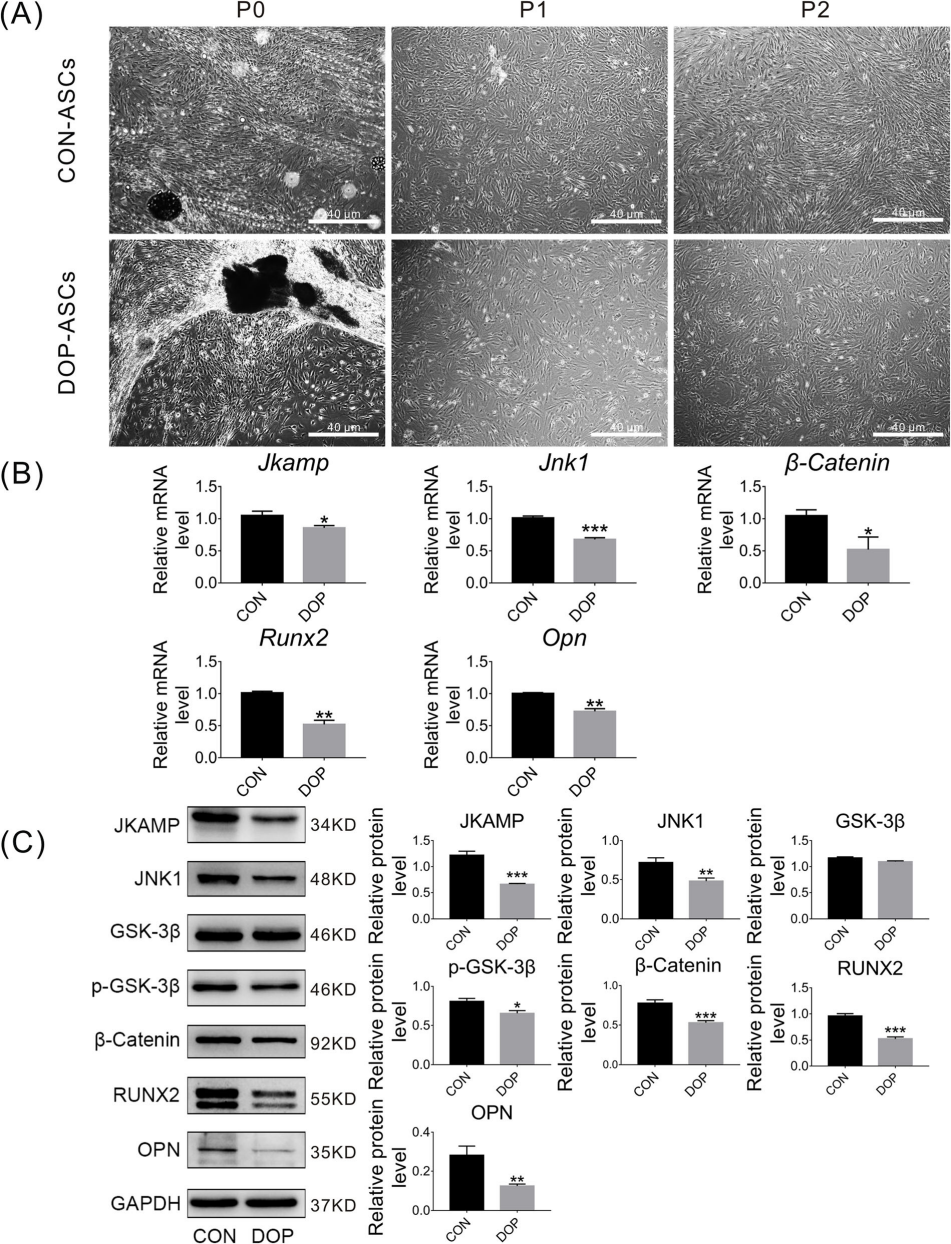
JKAMP, the Wnt signaling pathway, and osteogenesis-related molecules are suppressed in DOP-ASCs. **a** Normal appearance passage 2 CON-ASCs and DOP-ASCs. **b** mRNA levels of Jkamp, Jnk1, β-catenin, Runx2, and Opn in CON-ASCs and DOP-ASCs. **c** Protein levels of JAKMP, Wnt signaling pathway-related molecules, and osteogenesis-related molecules in CON-ASCs and DOP-ASCs. Data represent the mean ± SD of at least three independent experiments, *P < 0.05, **P < 0.01

To investigate differences in expression of JKAMP, Wnt signaling pathway markers, and osteogenesis-related molecules in CON-ASCs and DOP-ASCs, we cultured CON-ASCs and DOP-ASCs to passage 2 (Fig. 1a). After 3 days of osteogenesis induction, mRNA levels of *Jkamp*, *Jnk1*, *β-Catenin*, *Runx2*, and *Opn* were measured by qPCR. Moreover, western blotting was used to measure the protein levels of JKAMP, JNK1, GSK-3β, p-GSK-3β, β-catenin, RUNX2, and OPN. Compared with CON-ASCs, the gene and protein levels of Wnt signaling pathway markers and downstream osteogenesis-related molecules were reduced significantly in DOP-ASCs (Fig. 1b, c). These results showed that expression of JKAMP, the Wnt signaling pathway, and the osteogenic ability were downregulated in DOP-ASCs.

### JKAMP silencing suppresses the Wnt signaling pathway in CON-ASCs

**Fig. 2:**
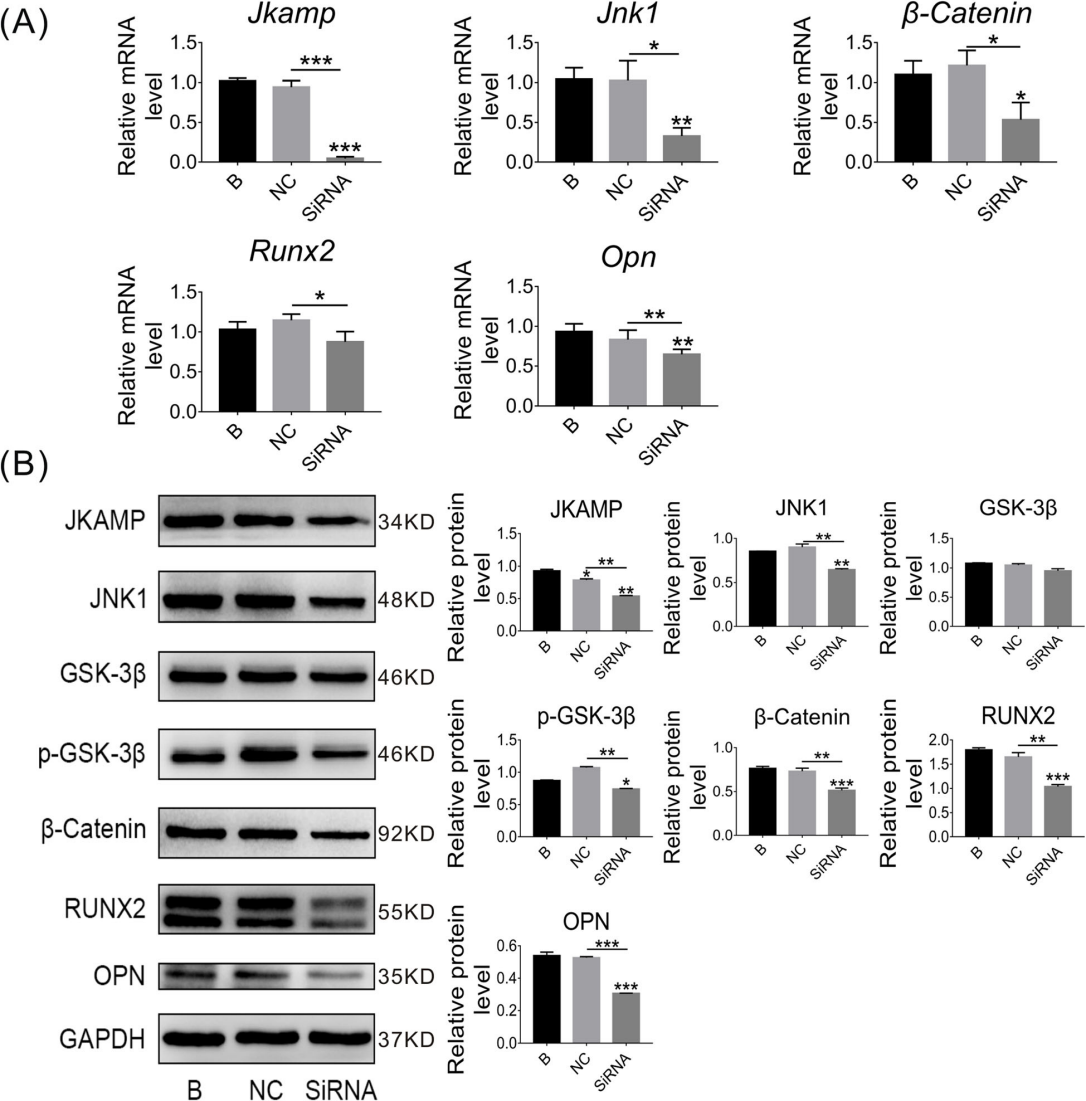
Si-*Jkamp* suppresses Wnt signaling and osteogenesis-related molecules in CON-ASCs (osteoinduction for 3 days). **a**, **b** Si-*Jkamp* downregulated the gene and protein levels of Wnt signaling pathway markers and osteogenesis-related molecules in CON-ASCs (osteoinduction for 3 days). Data represent the mean ± SD of at least three independent experiments, *P < 0.05, **P < 0.01

**Fig. 3:**
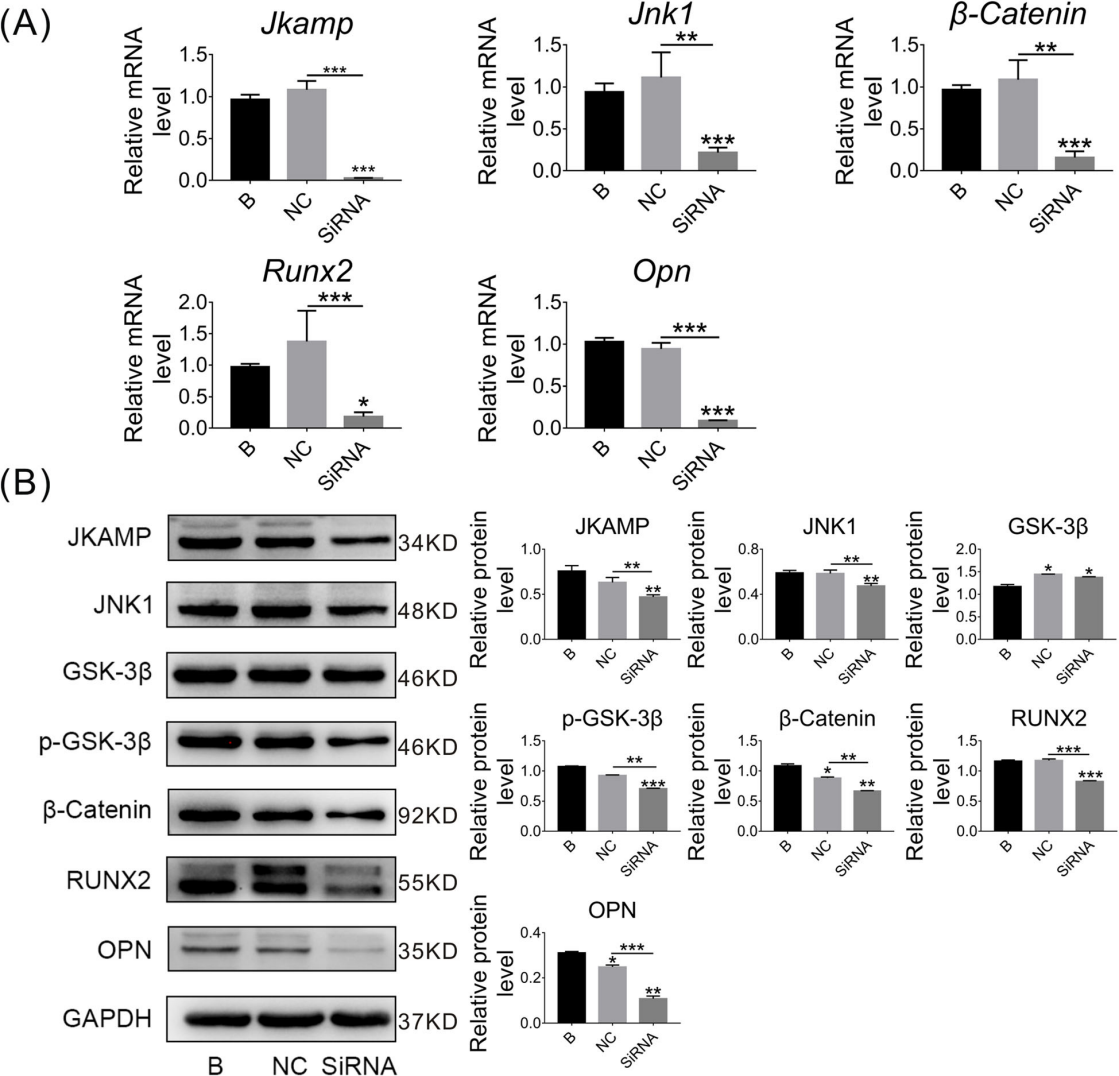
Si-*Jkamp* suppresses Wnt signaling and osteogenesis-related molecules in CON-ASCs (osteoinduction for 6 days). **a**, **b** mRNA and protein levels of Wnt signaling pathway markers and osteogenesis-related molecules were downregulated after si-*Jkamp* transfection into CON-ASCs (osteoinduction for 6 days). Data represent the mean ± SD of three or more independent experiments, *P < 0.05, **P < 0.01

JKAMP was highly expressed in CON-ASCs. However, the relationship between JKAMP and the Wnt signaling pathway in CON-ASCs was unclear. Therefore, we silenced the JKAMP gene in CON-ASCs and then detected expression of related genes and proteins in the Wnt signaling pathway. In the siRNA group, Si-*Jkamp* was used to silence *Jkamp*. Negative control (NC) and blank (B) groups were treated with the siRNA negative control and normal medium, respectively. The mRNA and protein levels of Wnt signaling pathway markers and downstream osteogenesis-related molecules in the SiRNA group were reduced significantly compared with B and NC groups after 3 days of osteogenic induction (Fig. 2a, b). On day 6 of osteogenesis induction, we obtained similar results (Fig. 3a, b). These results demonstrated that JKAMP and the Wnt signaling pathway were positively correlated.

### Osteogenesis of CON-ASCs cells decreases after JKAMP silencing

**Fig. 4:**
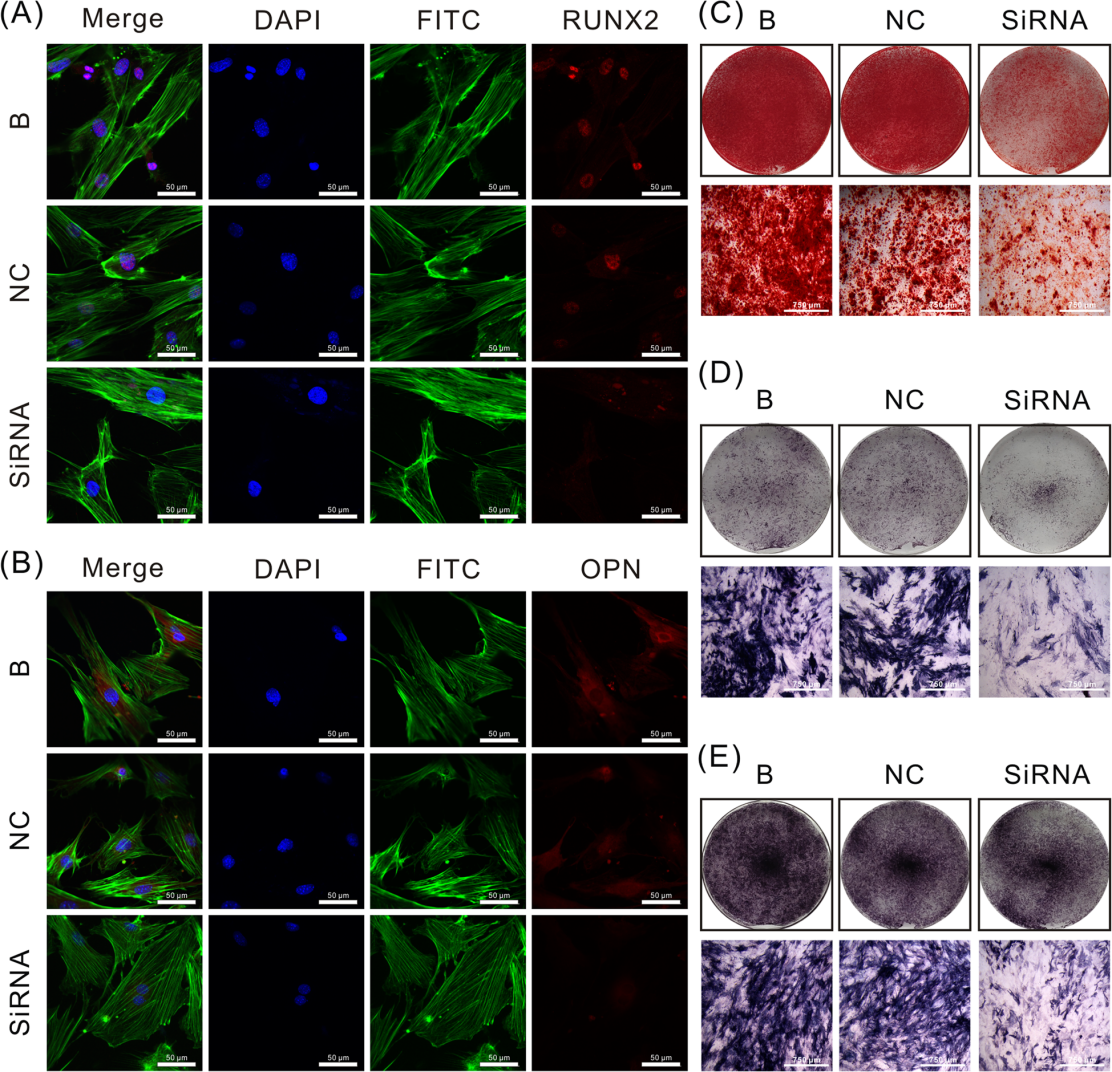
Si-*Jkamp* decreases the osteogenic ability of CON-ASCs. **a**, **b** Immunofluorescence staining of RUX2 and OPN proteins in CON-ASCs after 3 days after osteoinduction. **c** Alizarin red staining of CON-ASCs after 14 days of osteoinduction. **d**, **e** ALP staining of CON-ASCs after 3 and 5 days of osteoinduction. **d** Osteoinduction for 3 days. **e** Osteoinduction for 5 days

We used immunofluorescence, alizarin red, and ALP staining to determine changes in osteogenesis of CON-ASCs after *Jkamp* was silenced by Si-*Jkamp*. Immunofluorescence staining showed that OPN and RUNX2 proteins in the siRNA group were decreased after 3 days of osteogenic induction (Fig. 4a, b). After culturing the cells in osteogenic induction medium for 14 days, alizarin red staining revealed fewer mineralized nodules in the siRNA group compared with B and NC groups (Fig. 4c). ALP staining showed less alkaline phosphatase in the siRNA group compared with the other groups at 3 and 5 days after osteoinduction (Fig. 4d, e). These changes indicated that JKAMP positively regulated the osteogenic ability of CON-ASCs.

### JKAMP overexpressing activates the Wnt signaling pathway in DOP-ASCs

**Fig. 5:**
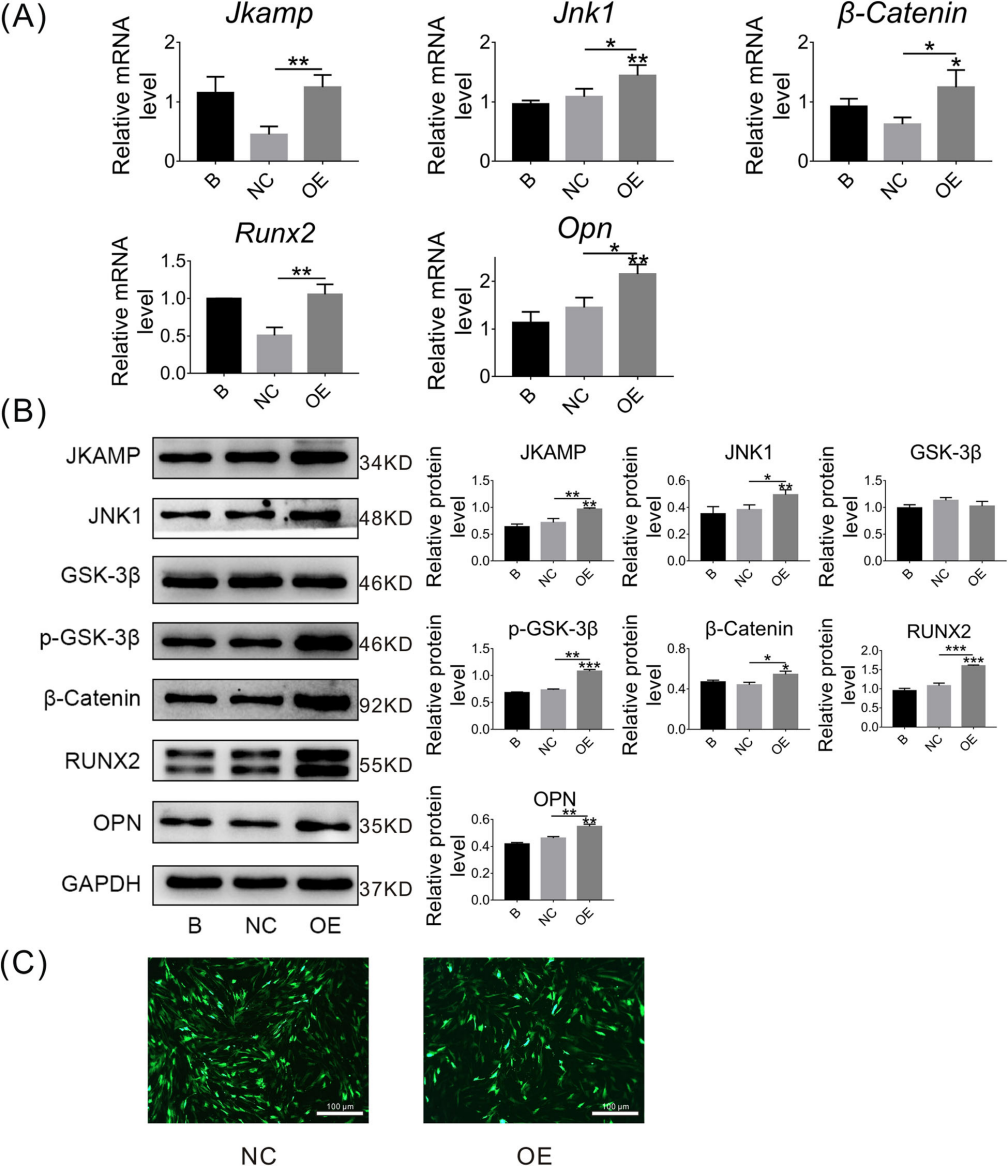
Overexpression of *Jkamp* increases Wnt signaling and osteogenesis-related molecules in DOP-ASCs (osteoinduction for 3 days). **a**, **b** The *Jkamp* plasmid upregulated mRNA and protein levels of Wnt signaling pathway markers and osteogenesis-related molecules in DOP-ASCs (osteoinduction for 3 days). **c** DOP-ASCs cellular uptake of NC- plasmids and OE- plasmids after treated for 48 h. Data represent the mean ± SD of at least three independent experiments, *P < 0.05, **P < 0.01

**Fig. 6:**
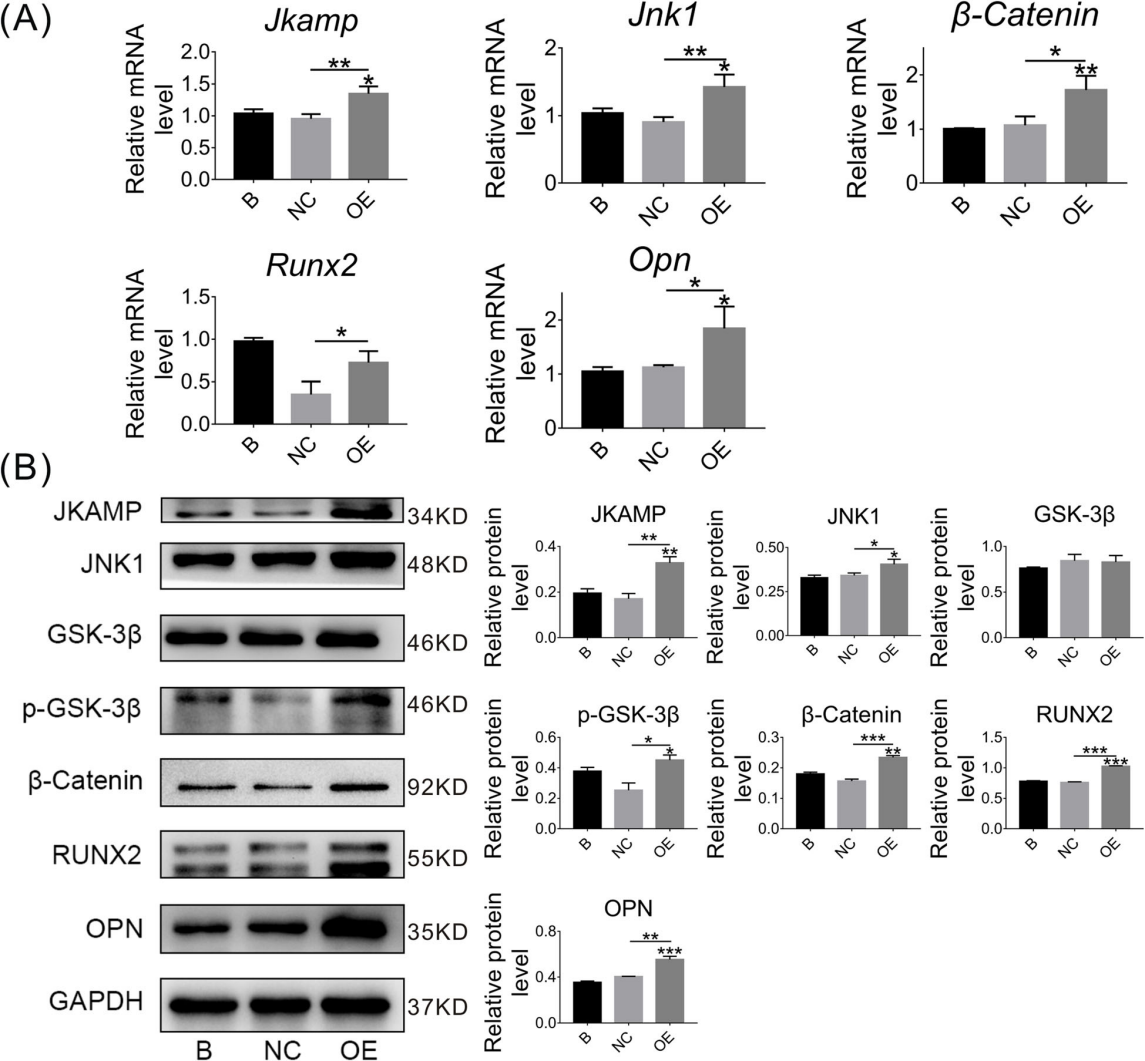
Overexpression of *Jkamp* increases Wnt signaling and osteogenesis-related molecules in DOP-ASCs (osteoinduction for 6 days). **a**, **b** mRNA and protein levels of Wnt signaling pathway markers and osteogenesis-related molecules were upregulated after *Jkamp* plasmid transfection into DOP-ASCs (osteoinduction for 6 days). Data represent the mean ± SD of at least three independent experiments, *P < 0.05, **P < 0.01

To activate the originally inactive Wnt signaling pathway in DOP-ASCs, we transfected an overexpression plasmid carrying the JKAMP gene into DOP-ASCs (Fig. 5c). Osteogenesis was then induced for 3 days after transfection. Compared with B and NC groups, DOP-ASCs transfected with the *Jkamp* plasmid (OE group) showed higher mRNA and protein expression of Wnt signaling pathway markers and downstream osteogenesis-related molecules (Fig. 5a, b). After osteogenesis induction for 6 days, similar results were obtained (Fig. 6a, b). These results indicated that overexpression of *Jkamp* activated the Wnt signaling pathway in DOP-ASCs.

### JKAMP overexpression increases osteogenesis of DOP-ASCs

**Fig. 7:**
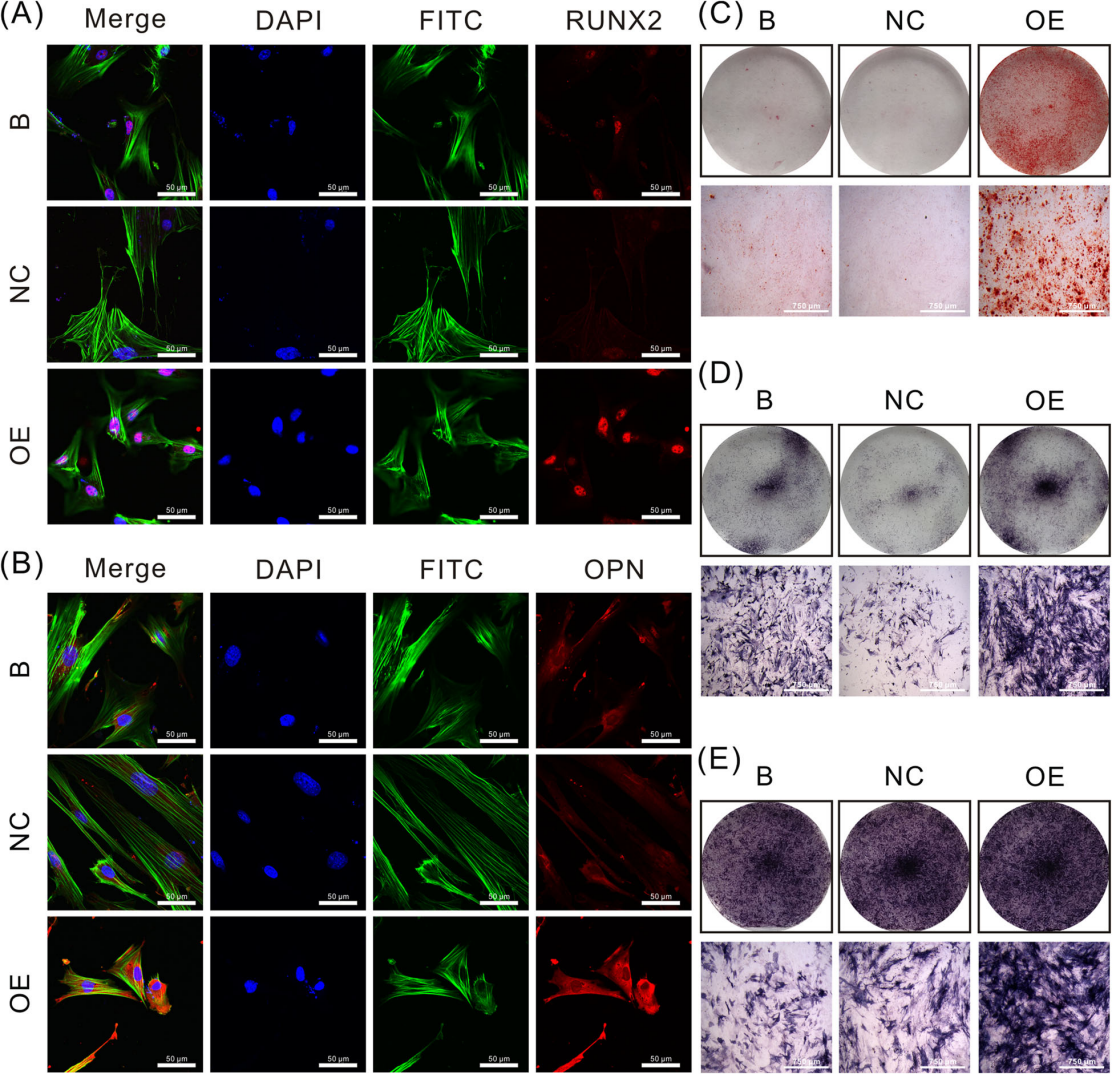
Overexpression of *Jkamp* increases the osteogenic ability of DOP-ASCs. **a**, **b** Immunofluorescence staining of RUX2 and OPN proteins in DOP-ASC after 3 days of osteoinduction. **c** Alizarin red staining of DOP-ASCs after 14 days of osteoinduction. **d**, **e** ALP staining of DOP-ASCs after 3 and 5 days of osteoinduction. **c** Osteoinduction for 3 days. **d** Osteoinduction for 5 days

Immunofluorescence, Alizarin red, and ALP staining were used to examine changes in the osteogenic ability of DOP-ASCs after *Jkamp* overexpression. Immunofluorescence staining showed that OPN and RUNX2 proteins in the OE group had increased compared with those in B and NC groups at 3 days after induction of osteogenesis (Fig. 7a, b). Alizarin red staining at day 14 of osteogenesis induction revealed that the OE group had more mineralized nodules than B and NC groups (Fig. 7c). ALP staining was performed at days 3 and 5 of osteogenesis induction, which showed that the OE group had more alkaline phosphatase activity than B and NC groups (Fig. 7d, e). These results showed that overexpression of *Jkamp* enhanced the osteogenic ability of DOP-ASCs.

### Increased intragenic methylation level of JKAMP in DOP-ASCs

**Fig. 8:**
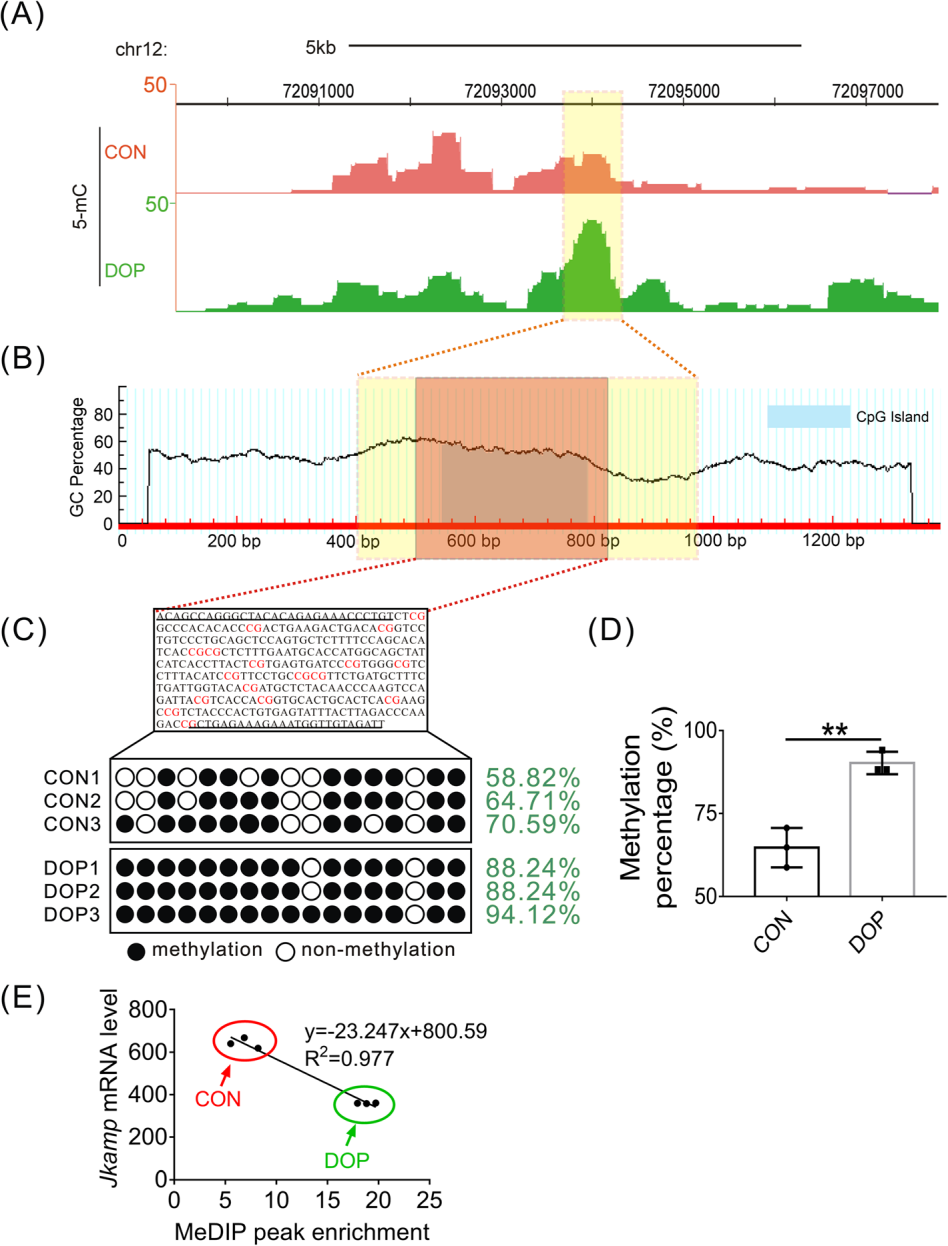
Increased methylation level of JKAMP in DOP-ASCs. **a** MeDIP sequencing showed that the intragenic methylation peaks of the JKAMP gene in DOP-ASCs were significantly higher than those in CON-ASCs. **b** Meth Primer software analysis showed that a large amount of CGI was enriched in the region near JKAMP exon 4 (genomic coordinates: chr12, 72093357–72094707). **c**, **d** BSP confirmed that the methylation degree of CON-ASCs was lower than that of matched DOP-ASCs in the region near exon 4 of JKAMP (genomic coordinates: chr12, 72093857–72094207). **e** Correlation between *Jkamp* mRNA levels and MeDIP peak enrichment near exon 4 of JKAMP (*r*^*2*^ = 0.977). Each data point corresponds to a high-throughput sequencing result, and the data were analyzed for correlation. Data represent the mean ± SD of at least three independent experiments, *P < 0.05, **P < 0.01

To explore the reasons for the decrease of JKAMP expression in DOP-ASCs, we performed MeDIP sequencing and BSP analyses of JKAMP in CON-ASCs and DOP-ASCs. Interestingly, MeDIP sequencing analysis revealed that the methylation peak of the JKAMP gene in DOP-ASCs was significantly higher than that in CON-ASCs, especially near exon 3/4/5/6/7 regions. However, there was no such difference in the promoter region (Fig. 8a). A recent study confirmed the existence of putative promoters near exons. Similar to promoter DNA methylation, putative promoter DNA methylation also individually regulates the expression of exons [33]. We hypothesized that expression of JKAMP was influenced by the CpG island (CGI) located at the gene body. Therefore, we examined the representative exon 4 region (gene coordinates: chr12, 72093937–72094143). Through calculation of Meth Primer software, we found a large amount of CGI enrichment in the region near exon 4 (genomic coordinate: chr12, 72093357–72094707) (Fig. 8b). BSP results also demonstrated that the degree of methylation of the genomic coordinate chr12 72093857–72094207 in DOP-ASCs was higher than that in CON-ASCs (Fig. 8c, d). Additionally, combined with the results of mRNA expression profiling and MeDIP sequencing, we found a strongly negative relationship between JKAMP expression and the methylation level of the corresponding CGI (Fig. 8e). Taken together, these observations indicated that the decrease in expression of JKAMP in DOP-ASCs was related to increased intragenic methylation.

## Discussion

The hyperglycemic microenvironment caused by diabetes exacerbates damage of osteoblasts. Chronic inflammation in diabetic patients inhibits the activity of stem cells and increases calcium excretion [1, 34, 35]. Additionally, the interaction of hyperglycemia with parathyroid hormone and the vitamin D system weakens bone turnover in diabetic patients and reduces osteocalcin produced by osteoblasts [36, 37]. The diabetic microenvironment makes bone tissue more susceptible to accumulation of microdamage, which leads to increased bone fragility and diabetic osteoporosis [2, 38, 39]. Recent studies have shown that DNA methylation is involved in multiple biological processes of stem cells. Zhang et al. found that transplantation of BMSCs modified by DNA methylation rescues the osteogenic ability in lupus mice [40]. Vera et al. reported that DNA methylation in a hyperglycemic environment changes the expression of CXCR4 receptors and migration of CD34+ stem cells [41]. However, at present, there are no reports of epigenetic changes in stem cells, which cause diabetic bone disease [38]. In this study, we established a mouse model of DOP and revealed the molecular mechanism by which JKAMP affects osteogenesis of DOP-ASCs via intragenic DNA methylation.

JKAMP is a seven-transmembrane protein that is mainly located in the plasma membrane of cells [42]. It provides cells with apoptosis-related signals and affects the activity and duration of JNK1 through competition with mitogen-activated protein kinase phosphatase 5 [20]. Sabapathy et al. reported that the main function of JNK1 is activation of c-Jun after stress, and JNK2 appears to regulate the stability of c-Jun under non-stressed conditions [43]. Boutros et al. reported that JNK1 interacts with Dsh to regulate the Wnt-PCP Pathway [44]. Another study has shown that JNK1 also cooperates with β-catenin to participate in the canonical Wnt pathway [22]. However, there is no study that has directly shown the relationship between JKAMP and the Wnt signaling pathway. In CON-ASCs, we found that silencing *Jkamp* decreased the expression levels of β-catenin and p-GSK-3β by reducing expression of JNK1. Furthermore, overexpression of *Jkamp* increased the expression levels of β-catenin and p-GSK-3β in DOP-ASCs by upregulating the expression of JNK1. In general, our results demonstrated that JKAMP positively regulated the Wnt signaling pathway through JNK1.

The Wnt/β-catenin signaling pathway plays an indispensable role in osteogenic differentiation and bone development of stem cells [45, 46]. Ubiquitination and degradation of β-catenin are regulated by the balance of GSK-3β and p-GSK-3β [22]. β-Catenin interacts with TCF/LEF-1 or other transcription coactivators in the nucleus and induces the expression of osteogenesis-related molecules to regulate osteogenesis [24, 25, 47]. Compared with CON-ASCs, DOP-ASCs exhibited relatively downregulated expression of β-catenin, p-GSK-3β, RUNX2, and OPN. These results indicated that the Wnt signaling pathway in DOP-ASCs was inhibited with a subsequent decline in osteogenesis. After silencing *Jkamp*, expression of Wnt signaling pathway markers in CON-ASCs was decreased, which decreased osteogenesis-related molecules over time. Additionally, after overexpressing *Jkamp* in DOP-ASCs, the levels of osteogenesis-related molecules were rescued by activating the Wnt signaling pathway. Interestingly, ALP and Alizarin red staining indicated that inhibiting the expression of JKAMP reduced the early and late osteogenic differentiation capacities of CON-ASCs. Conversely, overexpression of JKAMP rescued the early and late osteogenic differentiation capacities of DOP-ASCs. Considering that the changes in the osteogenesis-related molecules via Wnt signaling pathway after silencing or overexpression of *Jkamp* are consistent with the variations in ALP and Alizarin red staining, we have reason to believe that the osteogenic ability of ASCs was regulated by *Jkamp* and Wnt signaling pathway. Therefore, JKAMP inhibits the osteogenic capacity of DOP-ASCs by modulating the Wnt signaling pathway.

DNA methylation is covalent addition of a methyl group to the C5 position of a cytosine pyrimidine ring [48]. Generally, in a CpG dinucleotide, hypermethylation of CpG sites in a promoter leads to transcriptional silencing [49]. As an epigenetic marker, the main functions of DNA methylation include gene silencing and maintenance of genomic integrity. Additionally, it plays a vital role in genomic imprinting and suppression of repeated sequences [50–53]. Zhao et al. reported that promoter DNA methylation regulates the osteogenic differentiation ability of BMSCs from osteoporotic mice [54]. Zhang et al. showed that DNA methylation-related enzymes (DNMT1 and DNMT3a) are significantly upregulated in AGE-induced ASCs of diabetic models, which reduces osteogenesis [19]. In recent years, some studies have found that the role of intragenic methylation is seriously underestimated. In fact, intragenic and promoter methylations are both involved in transcriptional regulation of HIV-1 and reduce transcription efficiency of the virus [55]. Ma et al. found that putative promoters may exist in the gene bodies of certain cancer-related genes. DNA methylation of CGI near these putative promoters also silences these genes [32]. Mathios et al. found that intragenic DNA methylation of glioblastoma may also be a regulatory mechanism of ZMIZ1 gene transcription [56]. Interestingly, in our study, we found that the promoter of JKAMP showed no obvious difference in the DNA methylation degree between CON-ASCs and DOP-ASCs. However, the methylation degree of the CON-ASCs group was lower than that of the matched DOP-ASC group in the gene body, especially near exon 3/4/5/6/7 regions. Additionally, correlation analysis determined that the change in the JKAMP mRNA level was related to these intragenic methylations of the corresponding CGIs. These results suggest that the decreased expression of JKAMP in DOP-ASCs was related to intragenic DNA methylations rather than promoter DNA methylation.

Long non-coding RNAs (LncRNAs) are generally recognized as non-coding RNA molecules greater than 200 nucleotides in length [57]. Recent studies have postulated that they have a vital function in a series of important cellular processes [58, 59]. Moreover, growing evidence is supporting the involvement of LncRNAs in influencing gene expression by regulating DNA methylation of specific CGI [60]. Therefore, how LncRNAs interact with intragenic DNA methylations to affect the expression of JKAMP is a potential epigenetic mechanism that needs us to entail. And this research provides an epigenetic explanation for the reduced osteogenic ability of DOP-ASCs and a potential therapeutic target for the prevention and treatment of diabetic osteoporosis.

## Conclusions

This study shows that decreased expression of JKAMP reduces the osteogenic potential of DOP-ASCs by inhibiting the Wnt signaling pathway. Overexpression of JKAMP effectively rescues the decline in osteogenesis of DOP-ASCs. Furthermore, intragenic methylation of JKAMP in DOP-ASCs has a strong negative correlation with JKAMP expression, which provides a possible research route for bone tissue regeneration of diabetic osteoporosis.

## Data Availability

The datasets generated or analyzed during the current study can be obtained from the corresponding author in accordance with reasonable requirements.

## Abbreviations

DOP: Diabetic osteoporosis
DM: Diabetes mellitus
ASCs: Adipose-derived stem cells
CON: Control
BSP: Bisulfite-specific PCR
PCR: Quantitative real-time polymerase chain reaction
STZ: Streptozotocin
ALP: Alkaline phosphatase
Jnk1: c-Jun-N-terminal-kinase-1
JKAMP: JNK1\MAPK8-associated membrane protein
β-Catenin: Cadherin-associated protein
Runx2: Runt-related transcription factor 2
Opn: Osteopontin
GAPDH: Glyceraldehyde phosphate dehydrogenase
OE: Over-expressed
B: Blank
NC: Negative control
GSK-3β: Glycogen synthase kinase 3 beta
DNMT1: DNA methyltransferases 1
DNMT3a: DNA methyltransferases 3a
CGI: CpG island
LncRNA: Long non-coding RNA
